# A Critical Systematic Scoping Review on the Applications of Additive Manufacturing (AM) in the Marine Industry

**DOI:** 10.3390/polym17010004

**Published:** 2024-12-24

**Authors:** Ayberk Sözen, Gökdeniz Neşer

**Affiliations:** Institute of Marine Sciences and Technology, Dokuz Eylul University, 35340 Izmir, Türkiye; ayberk.sozen@deu.edu.tr

**Keywords:** additive manufacturing in marine industry, small marine craft building, 3D printing and related materials in marine use, technological forecast in shipbuilding

## Abstract

(1) Background: Additive manufacturing (AM), which has also become known as 3D printing, is rapidly expanding its areas of use in the marine industry. This study undertakes a historical development of AM in the marine industry. The study also criticises these developments to date and the future technological applications they will lead to, while considering the benefits for the industry and its segments. (2) Methods: This review followed the guidelines of the Preferred Reporting Items for Systematic Reviews and Meta-Analyses (PRISMA) and was registered in the Open Science Framework. The personalized search strategy was applied to Scopus, and Web of Science databases. The core emphasis was placed on two eligibility criteria throughout the evaluation process. Firstly, Criteria 1 sought to determine the paper’s relevance to AM. Secondly, Criteria 2 aimed to assess whether the paper delves into the implementation of AM or provides valuable insights into its utilisation within the marine industry. The risk of bias was analysed using a checklist of important parameters to be considered. (3) Results: In recent years, there has been a growing trend in studies related to the application of AM in the marine industry. While AM is widespread in industries such as automotive, aviation, and healthcare, it is relatively new for the marine industry. Almost only 5% of publications related to AM are related to the marine industry. There is a need for extensive research in many areas. It has been observed that classification societies and approval institutions, which largely drive the marine industry, have not yet taken AM into consideration sufficiently. (4) Conclusions: The studies show that AM is very promising for the marine industry. However, there are new studies at the experimental and theoretical level that need to be carried out to determine the right materials and AM methods to establish the quality control methodology and the necessary classification rules. This review also emphazises AM’s pivotal role in reshaping the marine industry, addressing the potential environmental and occupational safety effects of AM.

## 1. Introduction

Three-dimensional additive manufacturing, or three-dimensional printing, creates three-dimensional objects by adding material layer by layer from a digital model, contrasting with traditional subtractive manufacturing. AM uses various materials, such as plastics, metals, and ceramics, starting with a CAD-designed digital model. This method excels in producing complex geometries and customized parts by reducing material waste, with applications including scoping rapid prototyping, advanced manufacturing, medical implants, aerospace components, etc. Despite advantages like rapid production and design flexibility, AM faces challenges such as material limitations, size constraints, and the need for post-processing to improve surface finish [1].

Although the first notable examples of this technology were seen in the 1980s, AM has been used more extensively in the last decade due to its promising potential in designing structures with advanced materials due to increasing market competition, raw material and energy prices [2]. It should be emphasized that, particularly in the context of increasing demand for end-user-oriented personalized products, additive manufacturing specializes in fabricating complex geometries with rapid prototypes especially for low-quantity demands. Furthermore, in some advanced production with repair possibilities, profitability stands out [3,4].

**Figure 1:**
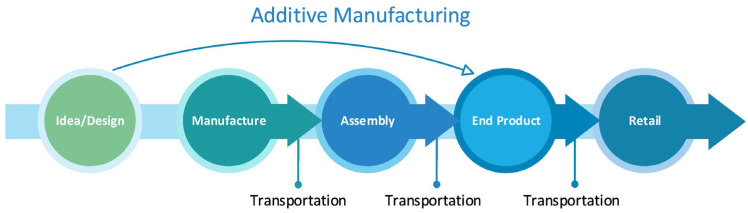
Contribution of AM to conventional supply chain.

The ability of AM to establish a direct connection with the designer and the end user, as seen in Figure 1, is vital today, where the supply chain can be broken because of the risks that are now part of life, such as pandemics and wars. In addition, due to the increasing market competition and demand, it is tough to find a large enough workforce for shipyards [5]. This technology, which will reduce the need for trained labour required for special productions, will also provide agility to the marine industry [6]. It is clear that many time-consuming intermediate stages, especially quality control, in the structure of the traditional supply chain divided into many processes will decrease.

AM is one of the fundamental aspects in achieving the goals of Industry 4.0 [7]. To this end, it can be said that AM aims to cost less, consume less energy, generate less waste, and, at the same time, have a more reliable production process. Secondly, cloud-based printers and designers communicate with each other using machine learning and integrate and develop production parameters and geometries with the support of artificial intelligence [8,9,10]. The design process for additive manufacturing is a longer and more iterative process that requires expertise compared to traditional manufacturing. Although this reduces the cost during production, it increases the design cost and duration [11]. AI-assisted designs are expected to resolve this situation.

**Figure 2:**
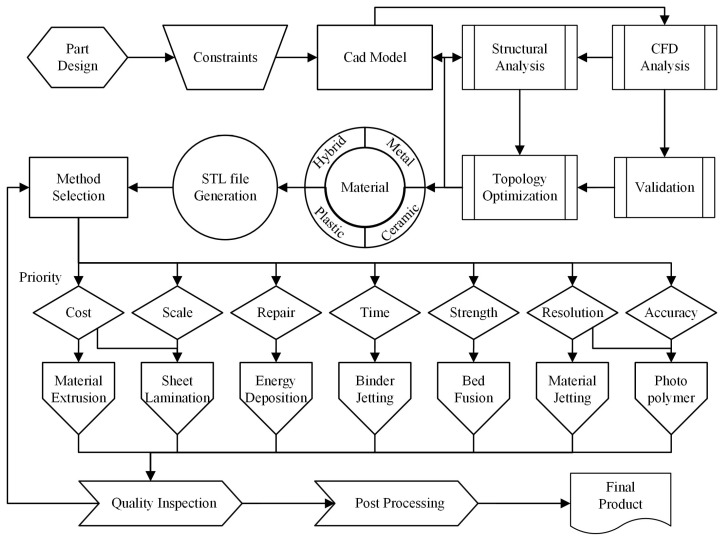
AM flow diagram of a potential product in marine use.

As summarized in Figure 2, AM begins with computer-aided design, which can be defined as modelling an object in a virtual environment as a vector, or a point cloud (mesh) structure. In addition, the design can be improved by optimising geometry and topology along with structural analysis. Then, this design will be translated into a triangular computer language with a cartesian coordinate system, namely the Standard Triangle Language (STL) file format. The STL format, used in 3D printing and CAD, represents 3D objects with a mesh of triangles. Available in ASCII and binary formats, it describes surface geometry but lacks colour and material details. Widely accepted for its simplicity, STL is essential in rapid prototyping, medical imaging, and engineering design despite sometimes producing large files. Although the STL format was developed in 1987, it is used widely today [12,13].

Material selection is crucial in manufacturing. Choosing a manufacturing method will depend on the specific needs and the geometry of the part to be produced. AM offers seven different production methods, each with its advantages and drawbacks. Ultimately, the decision will be based on the manufacturer’s priorities and the availability of materials. Then, by determining critical production parameters such as temperature, printing speed, table temperature, and filament diameter, the file can be used in numerically controlled production benches—utilizing either software provided by the printer manufacturer or, preferably, open-source software written in a basic language—which has been standard practice since the 1950s. [5,14]. A G-code-based data file developed for 3D printers uses this file from the computer with the help of a memory card or a data cable and applies it by processing this extensive information line by line [15]. Depending on the product size and dimension, this file can consist of tens of thousands of lines. Dealing with and debugging comprehensive information is a significant challenge for large parts, known as a “big data challenge”.

Following production, both quality control inspection and post-processing have significant importance. It is only when the product successfully clears these stages that it is ready for active use.

The new-generation printers can continue from where they left off when the electricity is cut off and automatically stop if there is a problem with the filament or raw material flow. They have also safety measures against overheating.

**Figure 3:**
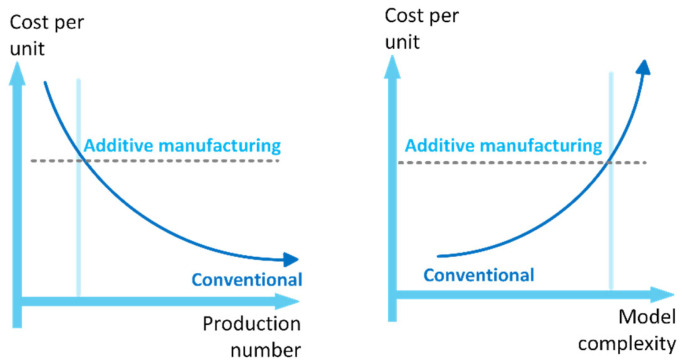
Comparison of AM with traditional manufacturing methods in terms of cost.

It is necessary to consider the parameters in the evaluation of the economic dimension of production with the AM. With today’s technology level, although AM shows significant changes according to the production techniques compared to the 1980s, the production cost is fixed, as shown in Figure 3. AM is preferable for products with complex forms and in low production quantities. On the other hand, when mass production is started, traditional production becomes advantageous in terms of cost, especially for products with simple forms. AM appears to be competitive, particularly in the “one-off” design segment such as marine recreational small-craft buildings [16,17].

A study compared the costs of injection molding and additive manufacturing for moderately complex automotive housings in 2017. Producing 20 parts via injection molding costs $120 per part, while additive manufacturing costs $20 per part and it is fixed. However, for over 200 parts, injection molding costs drop below $20 per part, and for 750 parts, costs fall below $10 per part due to the high mold cost of injection molding [18].

The main purpose of this review study is to present the research conducted so far on the applications of AM in the marine industry, to indicate the direction of the research in this area, and to determine future research needs. The review also aims to answer the following questions:

- How common is AM in the marine industry?
- What M techniques can be utilised in the marine industry?
- Why is the adoption of this technology less widespread in the marine industry compared to others?
- What are the parts with complex geometry that are difficult to produce in the marine industry?
- Which parts in the marine industry may be repaired using additive manufacturing?
- What are the potential benefits of AM in the marine industry?
- What could be the environmental impacts, occupational health hazards, and social effects of this technology?
- How can we adapt and disseminate AM in the marine industry?
- What additional research is necessary to improve the applications of AM in the marine industry?
- Would it be feasible to integrate intelligent materials into AM?

## 2. Materials and Methods

A systematic literature review was conducted to explore the information on the use of AM in the marine industry over 1996–2024. This comprehensive literature review, adhering to PRISMA (Preferred Reporting Items for Systematic Reviews and Meta-Analyses) guidelines, encompassed a total of 97 carefully chosen studies [19]. For this purpose, electronic databases, which are subscribed to by our university, were primarily used. Scopus and Web of Science were used as a primary search database.

### 2.1. Literature Search Strategy

**Figure 4:**
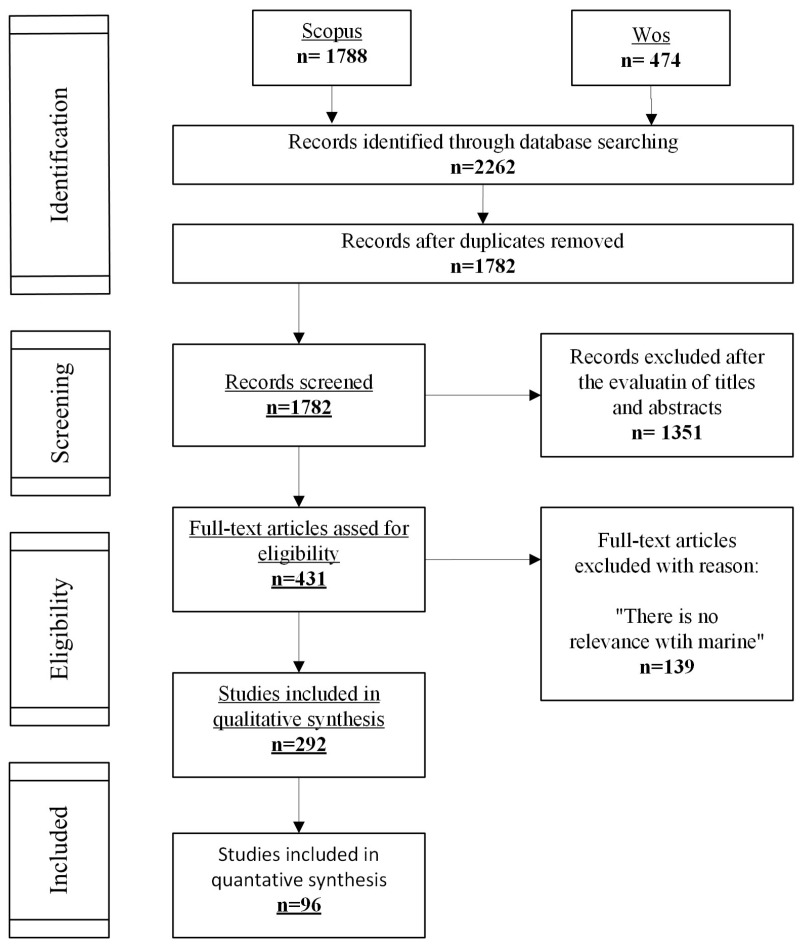
Systematic literature review process-flow diagram.

In the first step, a search was carried out using a combined two-part keyword system. The first set of keywords, referred to as context keywords, aimed to gather articles on AM and related subjects. The second level of keywords was designed to find papers that deal with the industry-wide applications of AM. To identify the articles that intersect these two levels, a dataset was created by combining the keywords from both levels. The specifics of the search process are outlined in Figure 4 [19,20,21,22].

The first set of context keywords related to AM included “3D printing”, “Additive manufacturing”, “Stereolithography”, “Selective laser sintering”, “Fused deposition modelling”, “3D printing technology”, “Additive manufacturing processes” and “3d printed materials”.

The second set of keywords targeting industry-wide applications, specifically in the marine industry, included “Marine applications”, “Shipbuilding”, “Naval engineering”, “Marine structures”, “Offshore platforms”, “Subsea equipment”, “Ship components”, “Ocean engineering” “Marine additive manufacturing” and “Naval architecture”.

### 2.2. Inclusion and Exclusion Criteria

The studies matching the following inclusion criteria were considered in this systematic review: “Is the paper related to AM?” and “Does the paper provide information on the use of AM in the marine industry?” Articles, reports, books, and other related documents indexed in Web of Science (WOS) and Scopus databases were included in the research.

In the next stage, duplicates were removed using the “VLOOKUP” formula in Excel (Version Office 365, 2024) software [20]. To ensure that the research only focused on AM in the marine industry, the abstracts of the remaining papers were then screened and grouped into four categories: unrelated fields, marginal relevance, closely related topics, and direct topics. The papers in the first two groups were excluded while those at the last two groups were included. For more precise analysis, a second screening was carried out on the abstracts to further categorise the papers into three groups: closely related concepts on AM but in other industries and application areas, tackling closely associated issues to AM at the marine industry level, and addressing concepts directly related to the topic. The first group was excluded, while the last two groups were included. Finally, a screening of the full papers was performed to determine which papers were related to the application of AM technologies in the marine field, which was the focus of the research [20].

### 2.3. Evaluation of Selected Studies

Following a meticulous assessment of both the title and abstract sections of the relevant publications, the full texts were read and evaluated. Publications that did not meet the inclusion criteria were excluded from this study. Any discrepancies regarding the inclusion or exclusion of a study were discussed among the authors until a consensus was reached. The data extraction form encompassed a variety of information for each study, including authors, year, country, journal, institution, department, marine industry segment, article type, and topic. In cases where studies were conducted in multiple institutes, the information pertaining to the first author of the article was documented in the data extraction form. Additionally, for the most frequently used terms, a word cloud was generated to provide insights.

To generate a word cloud from collected articles for the review using Python, several steps were taken. First and foremost, obtaining the article’s content and setting up essential libraries, such as *“wordcloud”* and *“matplotlib”,* were necessary to prepare the text. Afterwards, preprocessing the text was imperative, which involved converting it into lowercase, dividing it into words, and eliminating common stop words such as “the, and, of, etc…”. Next, determining the frequency of each word in the processed text was essential, which was achieved using Python’s “collections” library. Once completed, making the word cloud was initiated, allowing customisation of the final output’s appearance. Finally, the word cloud was displayed using *“matplotlib”* revealing the most frequent words in the article, with the size of each word being proportional to its frequency. This approach allowed us to quickly obtain an understanding of the key themes and trends within the text, making it a valuable asset for various purposes. The source code is given in Appendix A.

### 2.4. Risk of Bias Analysis

The evaluation of the study’s bias followed the methodology employed by Sarkis-Onofre [20], considering key parameters such as clarity in the Materials section, presence of suitable groups for comparison, and clarity in the Methodology section. Each parameter was scored as “Y” (Yes) if reported, and “N” (No) if not reported. The classification of bias risk was based on the number of parameters reported, with 6 or 5 items indicating low risk of bias, 4 or 3 indicating moderate risk, and 2 or 1 indicating high risk of bias [23].

## 3. Results and Discussion

### 3.1. Study Selection

The current search study yielded a cumulative total of 2262 records retrieved from the Web of Science and Scopus databases. Following the elimination of duplicate entries, 1782 records were retained for further evaluation based on their title and abstract content. Subsequently, a subset of 431 studies meeting the eligibility criteria underwent a comprehensive full-text assessment, and 89 studies were criticised for being included in this evaluation. The systematic delineation of the publication selection process is shown in Figure 4.

### 3.2. Scoping Synthesis of the Parameters

To understand the development stages of this technology, it is crucial to first look at the timeline of relevant publications by identifying the most productive countries and research centers in this field, as well as recognizing the publishers that have highlights these studies. This section presents the quantitative findings derived from the review.

According to research conducted on databases as mentioned, raw materials used in AM primarily focus on thermoplastic materials (28.2%), biomaterials (19.8%), and metals (13.5%). When questioned about the field, medicine ranks first (18.9%). Civil engineering covers 3.5% and the aviation and automotive industries cover 2.2%, while the marine industry covers only 0.4% of the literature. It can be said that additive manufacturing (AM) has been introduced to the marine industry only in recent years.

#### 3.2.1. Distribution of Publications by Years

**Figure 5:**
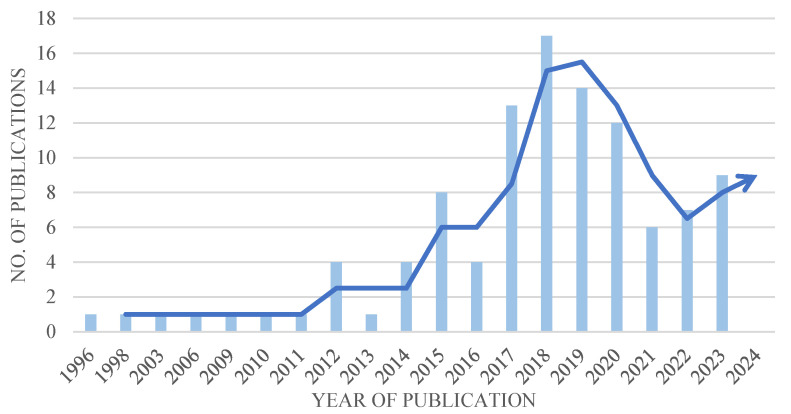
Distribution of the number of publications related to AM by years.

As shown in Figure 5, the publications analysed in the review are given by year, and it is clear that there has been a notable in studies related to AM in recent years, particularly after 2015. Despite a slight decrease in AM in the most recent year, it is important to note that this decline is related to the COVID-19 pandemic. Nevertheless, it is expected that research and development efforts will resume their upward trend soon. The marine industry is only beginning to reap the benefits of AM.

Recently, there has been a rise in theoretical and experimental studies exploring the use of AM in the marine industry [24]. The mentioned interest has been fuelled by the development of AM machines, which are capable of processing metal materials and producing larger products. Additionally, lower initial investment costs and improved supply chain efficiency have contributed to this trend [25].

Efforts are currently underway to bring additive manufacturing (AM) applications to the commercialization stage for the production of small marine crafts.. Despite this, it is still in the experimental stage. However, boats or their components with complex forms, such as propellers, are successfully produced, especially by using metals. AM, which has applications in many niche areas, such as producing artificial reefs and obtaining high-value-added upcycle products from the recycling of marine plastic, increases its potential to benefit the use of smart materials [14,26,27,28,29,30].

Smart materials are advanced materials that have the ability to respond dynamically to external stimuli such as temperature, pressure, moisture, pH, electric or magnetic fields, and light. These responses can include changes in their physical properties, such as shape, stiffness, viscosity, or colour, enabling them to perform specific functions. Examples include shape-memory alloys that return to a predetermined shape when heated, piezoelectric materials that generate electric charge in response to mechanical stress, and electrochromic materials that change colour with electrical input. The adaptability of smart materials makes them highly valuable in a wide range of applications, from medical devices and sensors to adaptive building materials and responsive textiles. Three-dimensional printing is a great tool for making complex shapes and personalized designs, and can be combined with smart materials [31,32,33].

#### 3.2.2. Distribution of Publications by Countries

**Figure 6:**
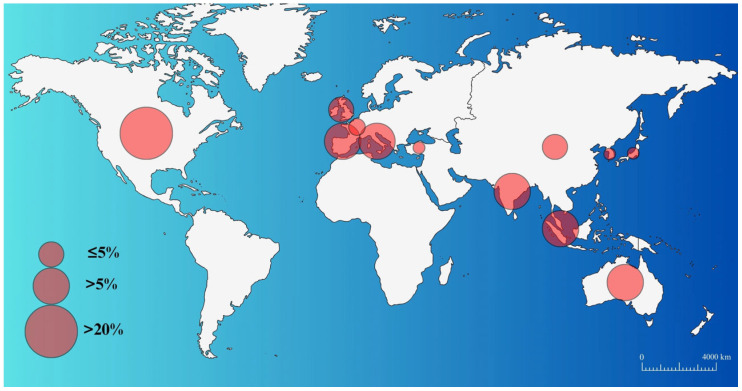
The representation of market shares of publications.

When examining the countries where research studies are conducted, it can be observed that the United States dominates with 28%, followed by Australia, India, Italy, Singapore, and Spain, each standing out with 6%. China and the United Kingdom are also contributors, each accounting for 5% of the conducted studies. An important aspect to be considered here is that although China holds a 5% market share, more than 20% of the published research is carried out by Chinese researchers from the United States and Europe as shown in Figure 6.

Upon reviewing publications, Additive Manufacturing, The Journal of Cleaner Production, Materials & Design, and Polymer are dominant journals in the area. An examination of the journals’ scopes showed that their topics of interest are strictly related to manufacturing processes and materials.

#### 3.2.3. Most Commonly Used Keywords

**Figure 7:**
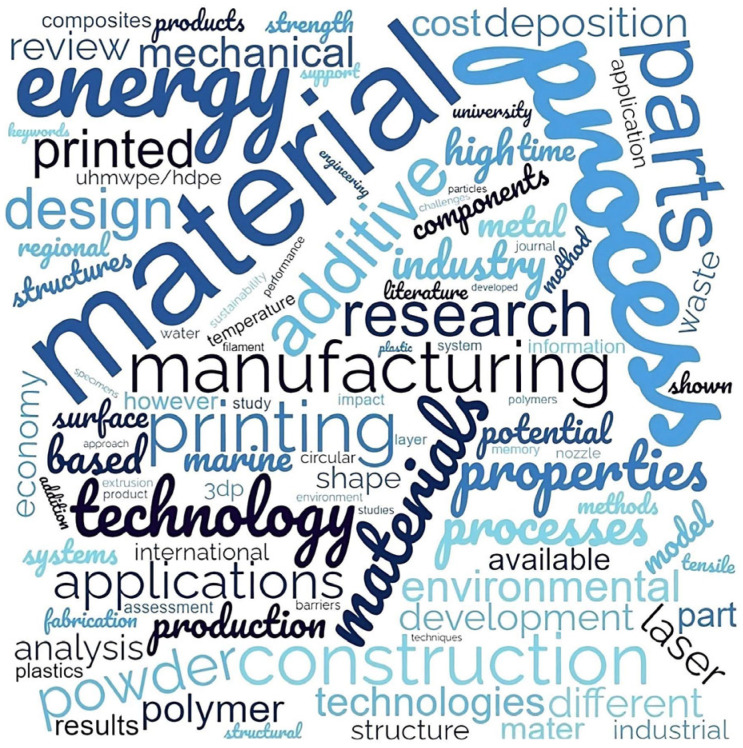
World cloud for AM articles surveyed.

In Figure 7, one can observe that certain words are commonly used in articles discussing current research topics. Common words are ‘material’, ‘method’, ‘energy’, and ‘properties’ (of printing). One area of focus is the amount of energy consumed during the production in AM. Additive manufacturing involves various processes and methods, and researchers are working on determining the optimal method based on factors such as part type and its complexity, quantity, size, and desired mechanical properties. Available materials for AM are limited, which has led to considerable research efforts to develop new materials, particularly new printable composites and smart materials.

### 3.3. Risk of Bias

Upon scrutinising the publications, it was determined that all of them carried a low risk, and no reportable situations were identified.

### 3.4. AM Technologies

AM can be divided into seven techniques, each with advantages and disadvantages. Table 1 presents these techniques along with the materials used, their precision levels, and associated costs. Choosing a proper technique depends on specific project requirements, such as material properties, part size and numbers, and targeted precision.

### 3.5. AM Techniques Used in Today’s Marine Industry

AM techniques for the marine industry are relatively new [41]. Unfortunately, it is not common to encounter awareness like in other sectors, specifically among marine small craft builders. In the conditions of increasing competition, small marine craft builders inevitably benefit from the design freedom, rapid prototyping and model production when using AM. Although it seems complicated to manufacture the whole boat from one piece at once, Maine University demonstrated in 2019 that it was possible to use polymer-based material. They broke the record by producing a 7.62 m–long boat in 72 h, excluding the material surface finishing process [42]. It was not possible to achieve this production speed with conventional manufacturing. As we mentioned before, the initial investment cost of AM is relatively high today. In addition, the surface of the product after printing needs finishing since a rough surface causes an increase in the resistance of the boat. Selecting suitable polymers is essential, such as high-density polyethylene (HDPE), polyethylene terephthalate glycol (PETG), and polyamide (PA), which are capable of withstanding outdoor conditions, or biodegradable materials coated with a protective layer could be used. Although biodegradable materials can be used for model production, petroleum-derived polymers, such as HDPE, PETG, and PA, are less risky for direct boat production and have proven their effectiveness in the marine environment.

Although there are precedents in producing the mold or the model for thermoplastic boat production in one piece, it requires a substantial investment. In addition, there is a risk of scrapping the entire model if damage occurs during production. Therefore, dividing production into parts and printing multiple sections will significantly reduce the initial costs. It is also relatively easy to replace a damaged part. In modular production, connection details are important. Mechanical and chemical bonding can be carried out, or polymer-based parts can be joined after printing with thermal welding. The 3D-printed part could also be considered as a core of a sandwich material and covered with fibre and resin thermoset face sheets. This assembly will both increase structural integrity and provide waterproofing in a marine structure. In 2020, Moi Composite Company (Milan, Italy) designed a boat named MAMBO (short for Motor Additive Manufacturing BOat). The boat's design includes negative angles in its form, which prevent it from being removed from the mold [17]. The production of this marine small craft has shown the potential of AM. The printed parts were joined by coating with a fibre-reinforced polymer. The Holland Shipyard Group is also working to produce autonomous electric passenger boats using the AM method [43].

It is important to note that most of these products are conceptual ideas. However, they offer design freedom. Additionally, this manufacturing method does not require molds, saving costs and simplifying production. This reduces initial setup costs and makes the process more flexible, improving productivity and responsiveness to market demands.

One of the advantages of polymer boat production with AM is that it contributes to reducing carbon footprint by recycling of the material. The research indicates that AM has potential cost savings of USD 170–593 billion, primary energy supply reductions of 2.54–9.30 EJ, and CO_2_ emission reductions of 130.5–525.5 Mt by 2025 [44]. According to another study focusing on wind turbines, 3D printing technology has been utilized to manufacture wind turbine systems with the aim of reducing the environmental impact associated with conventional construction methods. A comparison between conventionally manufactured components and those produced using 3D printing reveals a 25% reduction in CO_2_ emissions. This reduction is attributed to the shortening the supply chain, material phases, and transportation processes [44].

With the widespread use of polymers since the 1950s, a significant problem has arisen about how to manage their waste [45,46,47,48,49]. Caracol has produced a small-scale sailboat using plastic waste, with the help of AM technology. During production, a printer working with the material extrusion method was adapted to a robot arm used in the automotive industry [50].

AM is very common all over the world for hobby purposes, primarily based on polymer-based materials [34,51,52,53,54]. As a marine tradition, it plays a significant role in popularising ship modelling in naval schools. In ancient times, model craftsmanship required skills. Making models from simple ship drawings was not easy for beginners, but 3D printers made it easier. Interest in model-making with AM has increased. Users now only design, print, assemble and paint the mock-up. There is also a sport-orientated one-meter class designed for adults, where competitions are held both for sailboats and motorboats with remote control. Although this hobby attracts significant attention, these models are expensive despite their small size. AM makes manufacturing and customising these boats faster and at a lower cost. Another activity with remote-controlled boats is fishing. While a remote-controlled boat lays bait to attract fish, it tries to catch them with a fishing rod dangling from its stern.

Similarly, while the production of models for tank tests was carried out by hand in the past, computer-controlled benches are used today. With AM, manufacturing of these models will be faster and lighter by using fibre-reinforced polymer composites while models’ production costs are expected to decrease.

The prototype of the racing sailboat, which will be produced for the Youth America’s Cup, the youth-targeting organisation of America’s Cup, which is a well-established and essential organisation in the field of sailing, was made with AM in 2020. Sea trials were carried out in open water. Model testing or mathematical modelling of a foiling boat is complicated due to scale problems and sea conditions. Prototypes of such innovative designs need to be tested on a very realistic scale in actual sea conditions. Full-scale experiments, on the other hand, are costly. AM reduces the related costs for prototype production and allows more testing to be carried out. Furthermore, during the America’s Cup race in 2017, which featured catamaran boats, it became evident that the production of structural fittings for the Landrover team posed significant challenges when utilising traditional manufacturing methods. Renishaw has benefited from metal material-based AM for hull–hydraulic connections. Since America’s Cup is an innovative event and the races are held in an area closed to marine traffic, there is no standardisation for the boats and its elements [55].

In industrial uses, structural parts of autonomous underwater marine vehicles (AUVs) that dive to deep depths or autonomous surface vehicles (ASVs) that perform bathymetric work at the bottom of the sea, which are usually small in size, can be produced using polymer and metal materials by AM [56]. EcoSUB Robotic company (Camberley, UK) has manufactured a prototype of the critical parts with the power bed fusion technique of AM to produce AUVs [57]. In addition, AUV and ASV design competitions are well known in technology competitions held by universities as a contribution to engineering education.

Oak Ridge National Laboratory (ORNL) produced the mini-submarine prototype that will carry underwater personnel for the American Navy, with AM infrastructure [58]. This 5–6-month production, using traditional methods, was completed in just a few weeks. Production costs were reduced by 90%. It was stated that carbon fibre-reinforced composite was used as the printing material, but the matrix material was not specified. It was produced by joining relatively small-sized 3D-printed elements to each other.

Propellers, one of the most important components of a ship, are also of interest in AM. Damen Shipyard collaborated with the RAMLAB (Rotterdam, The Netherlands) and Autodesk software companies (San Francisco, CA, USA) to produce the first class-approved metal propeller in one piece [59]. Bureau Veritas supervised and approved the production as the classification society. Similarly, manufacturing boat parts such as valves, rudder, tiller, and even large engine crankshafts, etc., from metals is possible with AM. The machine elements that work under high thermal stresses are, therefore, subjected to heat treatments after printing. The French Navy experimentally produced 5-bladed propellers with a modular design using AM. This was also approved by Bureau Veritas (Paris, France) [60].

Most natural structures underwater are affected by marine pollution. Projects that try to improve marine habitats by creating artificial reefs are increasingly gaining momentum around the world. Sinking any obsolete aircraft or vehicle is no longer preferred today for this purpose. Ghost fishing nets have also negatively affected marine life. In this context, structures with complex geometries are needed to develop artificial reefs. Reefs are produced with large-scale polymer-based or cement 3D printers. Following these developments, AM in coastal engineering shows potential.

Additive manufacturing (AM) has great potential to improve the marine industry by enabling complex designs and lighter components as summarized examples in Table 2. However, there are several challenges associated with its use. These include limited material options, high costs, and difficulties in producing large parts. Environmental factors, like corrosion and biofouling, also affect the durability of AM parts, and the lack of standard certifications makes adoption slower. Table 3 briefly describes the current situation.

### 3.6. Research Needs

According to the literature survey conducted within the scope of this study, it was determined that there are research needs in the following areas:

- Material Development and Characterization: Investigating and developing new materials suitable for a sustainable marine environment such as especially recyclable thermoplastics, including corrosion-resistant metals, polymers, and composites. Conducting extensive material characterisation studies to ensure compliance with the related marine standards and regulations for strength, durability, and fire resistance.
- Process Optimization: Improving the speed and efficiency to reduce manufacturing time and costs. Carrying out studies on post-processing techniques to enhance surface finish and mechanical properties in parallel with these needs.
- Design of Products for AM: Developing guidelines and best practices for designing marine components specifically for AM, considering factors such as form complexity, weight reduction, topology optimisation and part consolidation.
- Quality Control, Standardisation and Certification: Establishing robust quality control procedures and certification standards for AM parts used in the marine industry. Investigating non-destructive testing (NDT) methods suitable for inspecting large, complex components.
- Cost–Benefit Analysis: Conducting cost–benefit analyses to evaluate the economic sustainability of AM for various marine applications, including shipbuilding, spare parts production, and repairs.
- Occupational Safety and Health: Investigation of the effects of AM processes and emissions on employees.
- Environmental Impact Assessment: Assessing the environmental impact of AM processes, including material usage and energy consumption, to ensure sustainability and compliance with marine environmental regulations.
- Marine-Specific Applications: Identifying and prioritising marine-specific applications for AM, such as propeller production, customised parts for marine interiors, and complex pipe systems.
- Supply Chain Integration: Studying the integration of AM into the marine supply chain to determine the most efficient way to manufacture, transport, and install AM components.
- Cybersecurity: Address cybersecurity concerns related to digital design files and 3D printing processes to protect against potential intellectual property conflicts.
- Education and Training: Developing curriculum and educational resources to train professionals to use AM technology effectively.
- Regulatory Framework: Collaborating with regulatory bodies to develop and update regulations and guidelines that address AM in the marine sector.
- Case Studies and Demonstrations: Conducting case studies and demonstrations to showcase the practical benefits of AM in the marine industry, including reduced lead times, cost savings, and improved performance.
- International Collaboration: Fostering international collaboration to share research findings, standards, and best practices in AM for the marine industry.

### 3.7. Special Topics and Future Outlook

In this section, possible future applications of AM in the marine industry will be discussed.

The use of smart/functional materials, which will increase the efficiency of the products throughout their life cycle, is becoming more common day by day [61,62]. These materials have limited applications in the industry, such as self-darkening windows due to sunlight and fire doors to warn operators by changing colours with temperature and luminous writings that glow at night. When the properties and functions of the materials that can be used with 3D printers change over time, these products are called 4D printed products. The 4th dimension in these materials is the time [63,64]. The use of memory-shaped materials, also known as smart materials, in this field seems possible in the near future. In this case, the material reacts and changes shape due to external stimuli such as humidity, temperature, light, electrical current, etc. [65,66,67,68].

**Figure 8:**
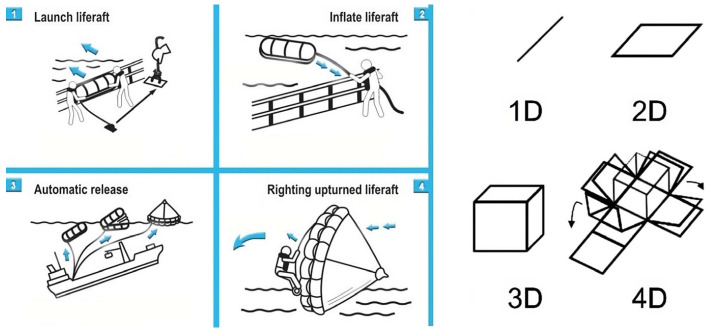
Self-expanding life rafts produced with smart materials by AM without inflation tube.

One of the areas where shape memory materials can be used in the marine industry is life rafts, as seen in Figure 8. Although it is easier on large ships to throw life rafts automatically into the water in case of danger, launching such a heavy object (approximately 40 kg) is quite difficult in a small marine craft with 8–9 people. Inflatable tubes, which constitute a significant part of the weight of life rafts, also have problems with inflating spontaneously in case of danger. For more reliable life raft production with smart materials, using AM seems to be a solution [69,70,71,72,73].

AM is more cost-effective than traditional methods, such as rotational molding, which rely on expensive metal molds for mass production of thermoplastic boats. Additionally, AM offers a more sustainable material option due to its recyclability. In thermoplastic molding production, AM can also be combined with metal materials, a process known as the hybrid method. This approach significantly reduces molding costs to more reasonable levels.

In addition, experimental studies of 5-axis printers are carried out today [74]. When 5-axis printers become widespread, the production speed will increase, and the capacity to process complex shapes such as ship forms will improve. This will have a positive impact on the cost–benefit ratio. The progress made in the manufacturing of metal and composite propellers is truly remarkable.

Since AM is controlled with many parameters, finding the optimum settings is tough. In this context, by using experimental results, the use of artificial intelligence can shorten the printing time, and the printing geometry and pattern can be redesigned by topology optimisation [36,75].

In addition, intensive work continues using thermosets in 3D printers [76]. If thermosets can be used cost-effectively in 3D printers, the small marine craft industry could come under a positive influence. Although thermoplastics are indispensable for low-cost 3D printers since they do not have molecular cross-links, their thermal stability is low, and their mechanical properties are moderate.

AM minimises human interference with the materials during production [77]. Emissions of polymer-based toxic substances with sub-micro-sized metal clouds during AM can adversely affect occupational health [77,78]. Even with a filtration system, nano-sized particulate materials pose a significant risk [79,80,81]. The presence of the HEPA filter system in the AM environment is critical [82]. Because this technology is relatively new, more research is needed on its long-term occupational health hazard [83,84]. Both the decrease in costs and the increase in the number of manufacturers, especially printers with VAT photopolymerisation and material extrusion technology, can be supplied by home users and small businesses. Inexperienced users face risks such as skin contact and burning with too hot material without protective personal equipment due to working in the same environment with open cabinet printers and exposure to toxic gases [38,85]. As of now, there are not any major limitations or attention given to these issues.

According to the life cycle assessment, studies determined that AM reduces the use of materials, accelerates production time, shortens the supply process, reduces the amount of waste, and reduces water consumption. However, it has been observed that AM increases energy use compared to conventional production, but different results have been obtained in further studies on energy consumption [86,87]. There is no consensus on energy issues in the AM field [88]. There is no comprehensive study specific to the marine industry. It is certain that energy consumption can be reduced by optimising the printing parameters and printing geometry [89,90]. Additionally, recycling waste parts with AM is relatively easy compared to standard production methods [91]. A thermoplastic boat manufacturer with this technology can produce new filaments and plastic welding wire from the thermoplastic waste within its ecosystem with a shredder and filament extrusion line.

AM technologies have yielded significant societal impacts, as noted by Huang [92]. Notably, advancements in the realm of medicine and implant technology have facilitated the reintegration of numerous individuals into society and have contributed to saving lives. The most significant impact is expected on workforce needs and dynamics. Conventional production methods traditionally rely on human labour at various stages within the supply chain, thereby generating employment opportunities [16,93]. The utilisation of AM allows for a more streamlined process, enabling designers to progress from raw materials to the final product with a reduced workload [6,94]. It is essential to recognise that this shift in labour dynamics can have unforeseen repercussions, particularly within industries like shipbuilding, which heavily depend on a skilled workforce [37].

Another important area for research involves the potential of 3D printers to replace conventional lathe and milling machines within the engine rooms of large ships. This requires a comprehensive analysis of the cost–benefit dynamics. While the initial investment in a 3D printer may appear substantial, the expense associated with a ship’s downtime and operational disruptions for the shipowner can be even greater. Moreover, it is impractical to stockpile every conceivable spare part.

This approach offers the possibility of on-the-spot ship repairs in numerous scenarios, mitigating the need to await spare part deliveries at sea. However, it raises questions about the suitability of printers for operation within the challenging environment of engine rooms, characterised by vibrations and limited ventilation. The feasibility of 3D printers functioning effectively in such conditions requires further investigation and assessment.

Replacing older, discontinued spare parts for maintenance poses a significant challenge to the longevity of marine equipment especially for offshore renewable energy structures [95,96]. The automotive industry has tackled a similar challenge by using AM. The aerospace and automotive sectors have successfully established standardised practices and documentation for AM. In contrast, the marine industry has been somewhat conservative in accepting this technology, primarily due to its lack of experience and confidence in its application.

In today’s rapidly changing economic and societal environment, supply chain processes can be fragile, while recruiting skilled personnel can also be a hard task. Supply chains are uncertain because they may be disrupted by geopolitical conflicts or global pandemics. These disruptions could cause problems in production. AM offers more confidence between the design and the final product.

## 4. Conclusions

To conclude, this comprehensive systematic review has provided a multifaceted exploration of additive manufacturing (AM) applications within the marine industry. It focused on the historical evolution of this transformative technology and examined its current impact on various segments, from ship design and building to maintenance and sustainability practices.

AM techniques that can be utilized in the marine industry include 3D printing, selective laser sintering (SLS), and direct energy deposition (DED), which enable the creation of complex, customized parts that would be difficult to produce using traditional methods.

In the marine industry, parts with complex shapes, such as propellers, hull structures, and internal components of engines, are difficult to make using traditional methods. These parts often have amorphous surfaces, internal cavities, or detailed designs that are hard to achieve with conventional manufacturing techniques. For example, propellers with specific shapes for different types of ships, or internal parts like cooling systems with precise channels, could be challenging to produce. Additive manufacturing solves these problems by allowing these complex parts to be made directly from digital designs.

Throughout the analysis, it has been observed how AM has not only revolutionised traditional manufacturing processes but has also fostered innovation and efficiency in the marine industry. The cases presented here have underscored the successful implementations of AM, demonstrating its potential to enhance both economic and environmental sustainability in the industry.

However, the review has also highlighted that while AM holds immense promise, it also includes challenges. Issues related to material selection, quality control, and regulatory considerations remain areas that require ongoing attention and research. As the marine industry continues to evolve, AM must adapt to address these challenges and seize new opportunities.

Looking ahead, the future of AM in the marine industry appears promising. As the technology matures and our understanding of its uncertainties increases, we can anticipate even greater innovations and advancements. These may include the development of novel materials tailored specifically for marine applications, further automation of the production process, and continued efforts to reduce environmental footprints through sustainable practices.

In closing, this review underscores AM’s pivotal role in reshaping the marine industry. It serves as a call to action for researchers, industry professionals, and policymakers to collaboratively address the challenges and harness the opportunities presented by this transformative technology. With concerted efforts and ongoing research, AM has the potential to revolutionise the marine industry by increasing efficiency, cost-effectiveness, and environmental responsibility.

## Figures and Tables

**Table 1:** AM techniques [5,10,25,34,35,36,37,38,39,40].

Methods	Description	Materials	Advantages	Limitations	Dominant Sector	Marine Application
Vat photo-polymerisation	Selective exposure to light, facilitated by a laser or projector, is employed to cure a reservoir containing liquid photopolymer resin. This process triggers polymerization and transforms the illuminated regions into a solid component.	UV-curable photopolymer resins	High-precision, smooth surface finish, biocompability	Limited build volume, material limitations, post-curing requirements, limited material recycling.	High resolution and surface finish, making it suitable for applications that require detailed and precise parts, such as jewelry, dental, and medical industries.	May not be suitable for all marine applications, especially require high mechanical strength. However, for prototyping, customized components, tooling, molds, and model making, it can provide significant benefits in terms of speed, accuracy, and design flexibility in the marine industry.
Material jetting	Parts are created by depositing material droplets layer by layer. This can be performed through methods such as jetting a resin that cures under UV light or jetting thermally molten materials that solidify at room temperature.	Plastics, metal and ceramic powders, and sand	High resolution, multi-material capability, good surface finish.	Expensive equipment, limited build volume, post-processing required.	High-resolution printing and the ability to use multiple materials in a single print job. It is utilized in industries such as dental, jewelry, and prototyping.	While material jetting has advantages in terms of high resolution and multi-material capabilities, it may not be suitable for applications that require high mechanical strength or durability in harsh marine environments. However, for prototyping, customization, visualization, mold production, and small part production, material jetting can offer significant benefits in the marine industry.
Binder Jetting	Thin layers of powdered material are built up layer by layer by selectively applying liquid bonding agents. These binders can be made of organic or inorganic materials. After printing, metal or ceramic powdered parts are usually fired in a furnace.	Powdered plastic, metal, ceramics, glass, and sand	Fast printing speed, large build volume, multi-material capability.	Lower resolution, post-processing required, lower strength compared to other methods.	Gaining traction in industries where high-speed production and large-scale parts are required. It has potential applications in automotive, aerospace, and architecture industries.	While binder jetting offers advantages in terms of design flexibility and customization, it may not be suitable for applications that require high mechanical strength However, it is ideal for casting patterns, customized components, tooling, lightweight structures, prototyping, and repair applications.
Material extrusion	Material is pushed out through a nozzle in the form of filaments, which are subsequently joined together to create multi-layer models. This process is commonly seen in heated thermoplastic extrusion.	Thermoplastic filaments	Low-cost printers, wide material selection, simple process.	Lower resolution, visible layer lines, limited strength and heat resistance.	Popular and widely accessible method, often used for prototyping and hobbyist applications due to its lower cost and ease of use.	While material extrusion offers advantages such as affordability and ease of use, it may not be suitable for applications that require high mechanical strength, high-temperature resistance, or excellent surface finish. However, it can be used for prototyping, concept models, and educational purposes.
Power bed fusion	The process of selectively consolidating powdered materials involves melting them together using a heat source like a laser or electron beam. During this process, the powder surrounding the consolidated part acts as support material for any overhanging features.	Plastics, metal and ceramic powders, and sand	Suitable for complex metal parts, good mechanical properties, no support needed.	Expensive equipment, limited material options, requires specialized safety measures.	Widely used in metal additive manufacturing for aerospace, automotive, and medical industries, producing parts with excellent mechanical properties.	Powder bed fusion provides significant advantages in terms of design freedom, material options, and production capabilities for marine applications. However, factors such as cost, build volume limitations, post-processing requirements, and material properties should be considered when evaluating its suitability for specific marine projects.
Sheet lamination	Layers of material are stacked and fused together through various lamination methods, such as adhesives or chemicals for paper/plastics, ultrasonic welding, or brazing for metals. Once built, excess portions are carefully removed layer by layer to achieve the desired shape.	Paper, plastic sheets, and metal foils/tapes	Cost-effective, large build volume	Limited resolution, mechanical properties, design complexity.	Produces larger parts and prototypes at lower costs, used in architecture, product design, and entertainment.	While sheet lamination offers advantages in terms of prototyping, large-scale modeling, tooling, and customization, it may not be suitable for applications that require high mechanical strength or durability in harsh marine environments.
Directed energy deposition	Powder or wire is introduced into a molten pool that forms on the surface of the part. The material then bonds to the underlying layers or part by utilizing an energy source like a laser or electron beam. This process can be seen as a type of automated build-up welding.	Metal wire and powder, with ceramics	Suitable for large-scale applications, repairs, and multi-material deposition.	Limited resolution, surface roughness, higher cost compared to some other methods.	Used for repair and modification of existing parts, as well as the production of large-scale metal components used in industries such as aerospace and defense.	It offers advantages such as repair capabilities, large-scale production, customization, and material versatility in the marine industry. However, factors such as process complexity, post-processing requirements, and material properties should be considered.

**Table 2:** Classification of marine industry application of AM techniques.

	Material Extrusion	Power Bed Fusion	Direct Energy Deposition	Sheet Lamination	Binder Jetting	Material Jetting
Artificial reef	X					
Mock-up	X					
Propeller	X	X	X			
Auxiliary machinery			X			
Engine part			X			
Boat hull	X					
Boat mold				X	X	X
Boat accessories	X		X		X	X
Interior design object	X					

**Table 3:** Unlocking opportunities and overcoming challenges.

Category	Problem	Impact	Potential Solutions
Material Performance	Limited availability of materials suitable for marine environments	Corrosion, biofouling, and reduced lifespan of AM parts	Development of corrosion-resistant and marine-grade AM materials
Cost	High cost of AM equipment and raw materials	Limited adoption in small-scale or cost-sensitive marine applications	Investment in scalable, cost-efficient AM technologies
Structural Integrity	Difficulty in achieving the required strength and durability for large-scale marine components	Potential failure under high pressure and harsh marine conditions	Optimization of design and material selection
Size Constraints	Limitations in AM machine build size for large marine components	Inability to produce large parts like ship hull sections or propellers	Hybrid manufacturing approaches and modular design strategies
Post-Processing	Complex post-processing requirements for marine components	Increased production time and costs	Automation and streamlining of post-processing techniques
Surface Quality	Poor surface finish of AM parts	Increased risk of drag and biofouling in marine applications	Improved AM techniques and coating technologies
Certifications	Lack of standardized certifications for AM parts in marine applications	Slower adoption	Development of industry-specific certification frameworks
Environmental Factors	Exposure to saltwater, UV radiation, and extreme temperatures	Accelerated wear and tear of AM-produced components	Testing and validation of AM materials for harsh environments
Design Expertise	Limited expertise in designing for AM in marine applications	Suboptimal designs that fail to leverage AM’s full potential	Training programs and design tools tailored for AM in marine contexts
Adoption Rate	Resistance to adopting new technologies in a traditional industry	Slower integration of AM into marine manufacturing	Education and demonstration of AM’s long-term benefits

## Data Availability

All references have been taken mostly from journal articles, and data are publicly available on the Web of Science and Scopus databases. The World cloud code has been shared in the Appendix A section.

## Appendix A. Python Code for Word Cloud

with open(“article.txt”, “r”, encoding=“utf-8”) as file:

article_text = file.read()

pip install nltk wordcloud

import nltk

from nltk.corpus import stopwords

from nltk.tokenize import word_tokenize

nltk.download(“stopwords”)

nltk.download(“punkt”)

# Lowercasing and tokenization

words = word_tokenize(article_text.lower())

# Removing stopwords

stop_words = set(stopwords.words(“english”))

words = [word for word in words if word not in stop_words]

from collections import Counter

word_freq = Counter(words)

from wordcloud import WordCloud

import matplotlib.pyplot as plt

wordcloud = WordCloud(width=800, height=400, background_color=“white”).generate_from_frequencies(word_freq)

plt.figure(figsize = (10, 5))

plt.imshow(wordcloud, interpolation = “bilinear”)

plt.axis(“off”)

plt.show()
